# Challenges and Current Status of the Biological Treatment of PFAS-Contaminated Soils

**DOI:** 10.3389/fbioe.2020.602040

**Published:** 2021-01-07

**Authors:** Esmaeil Shahsavari, Duncan Rouch, Leadin S. Khudur, Duncan Thomas, Arturo Aburto-Medina, Andrew S. Ball

**Affiliations:** ^1^School of Science, RMIT University, Bundoora, VIC, Australia; ^2^ARC Training Centre for the Transformation of Australia's Biosolids Resource, RMIT University, Bundoora, VIC, Australia

**Keywords:** PFAS-contaminated soils, bioremediation, mycoremediation, bioaccumulation, bacteria, phytoremediation

## Abstract

Per- and polyfluoroalkyl substances (PFAS) are Synthetic Organic Compounds (SOCs) which are of current concern as they are linked to a myriad of adverse health effects in mammals. They can be found in drinking water, rivers, groundwater, wastewater, household dust, and soils. In this review, the current challenge and status of bioremediation of PFAs in soils was examined. While several technologies to remove PFAS from soil have been developed, including adsorption, filtration, thermal treatment, chemical oxidation/reduction and soil washing, these methods are expensive, impractical for *in situ* treatment, use high pressures and temperatures, with most resulting in toxic waste. Biodegradation has the potential to form the basis of a cost-effective, large scale *in situ* remediation strategy for PFAS removal from soils. Both fungal and bacterial strains have been isolated that are capable of degrading PFAS; however, to date, information regarding the mechanisms of degradation of PFAS is limited. Through the application of new technologies in microbial ecology, such as stable isotope probing, metagenomics, transcriptomics, and metabolomics there is the potential to examine and identify the biodegradation of PFAS, a process which will underpin the development of any robust PFAS bioremediation technology.

## Introduction

As a result of continued production and use, per- and polyfluoroalkyl substances (PFAS) have become widespread in the environment, including drinking water, rivers, groundwater, wastewater, household dust, and soils (Kim et al., 2007; Eriksson and Karrman, 2015; Shi et al., 2015; Eriksson et al., 2017; Von Der Trenck et al., 2018). PFAS are highly stable organic compounds that contain multiple carbon-fluorine bonds. They are used in various commercial products, including aqueous fire-fighting foams and products with non-stick coatings. These compounds are also likely to be present in foods (Schaider et al., 2017) and are known to be present in humans, including pregnant women (Lauritzen et al., 2016).

Human exposure to PFAS occurs through several pathways, including ingestion of contaminated drinking water, food and household dust, inhalation of indoor air, and contact with other contaminated media (Trudel et al., 2008). Drinking water sources include rivers, lakes and groundwater may also be contaminated with PFAS originating from industrial sources. There may also be significant exposure risk from PFAS-contaminated sewage sludge (biosolids) and recycled water from wastewater treatment plants, which are often used in agriculture, with exposure through contaminated soils and crop foods (Sunderland et al., 2019). PFAS have been shown to have bioaccumulation potential, which tends to increase with increasing chain length. Specific PFAS compounds have been shown to impact human health through altered kidney and thyroid function, immunosuppression and deleterious effects on reproduction and development. Perfluorooctane sulfonate (PFOA)-related chronic diseases include kidney and testicular cancers, ulcerative colitis, and high cholesterol have also been observed (Darrow et al., 2013; Steenland et al., 2013; Starling et al., 2017; Sunderland et al., 2019). PFOS and PFOA are readily absorbed through the gut and are not metabolized, meaning body loads become excessive before they are excreted. PFAS are believed to act as endocrine disruptors through alterations in estrogen- and androgen-receptor functions (Mora et al., 2017). Research conducted by Tao et al. found that PFOS and PFOA accumulate in the serum of adults and blood of newborn babies, which indicates that breast milk is a major pathway for transferal (Tao et al., 2008a,b). Research indicated that the milk of mothers who have given birth to multiple children tend to have slightly higher levels of PFAS (Mora et al., 2017).

PFAS are considered to be stable and amphiphilic, exhibiting both hydrophobic and lipophobic tendencies (Giesy et al., 2010) resulting in ready accumulation within lipids (fats) and proteins (Mora et al., 2017; Seo et al., 2018). Based on the above health issues, there is an urgent need to remove these compounds from soils. Current methods to remove PFAS from contaminated soils are expensive, impractical for *in situ* treatment, use high pressure and temperatures, and/or result in toxic waste. Biodegradation has the potential to form the basis of a cost-effective, large scale *in situ* remediation strategy for PFAS. However, information about the biodegradation of PFAS by fungal and bacteria is limited. Consequently, this review aims to review chemical properties, the source of PFAS contamination in soils and summarize the remediation technologies, focussing on the potential of bioremediation for the safe and effective removal of PFAS from soils.

## Chemical Properties of Per-and Polyfluoroalkyl Substances (PFAS) Components

**Figure 1:**
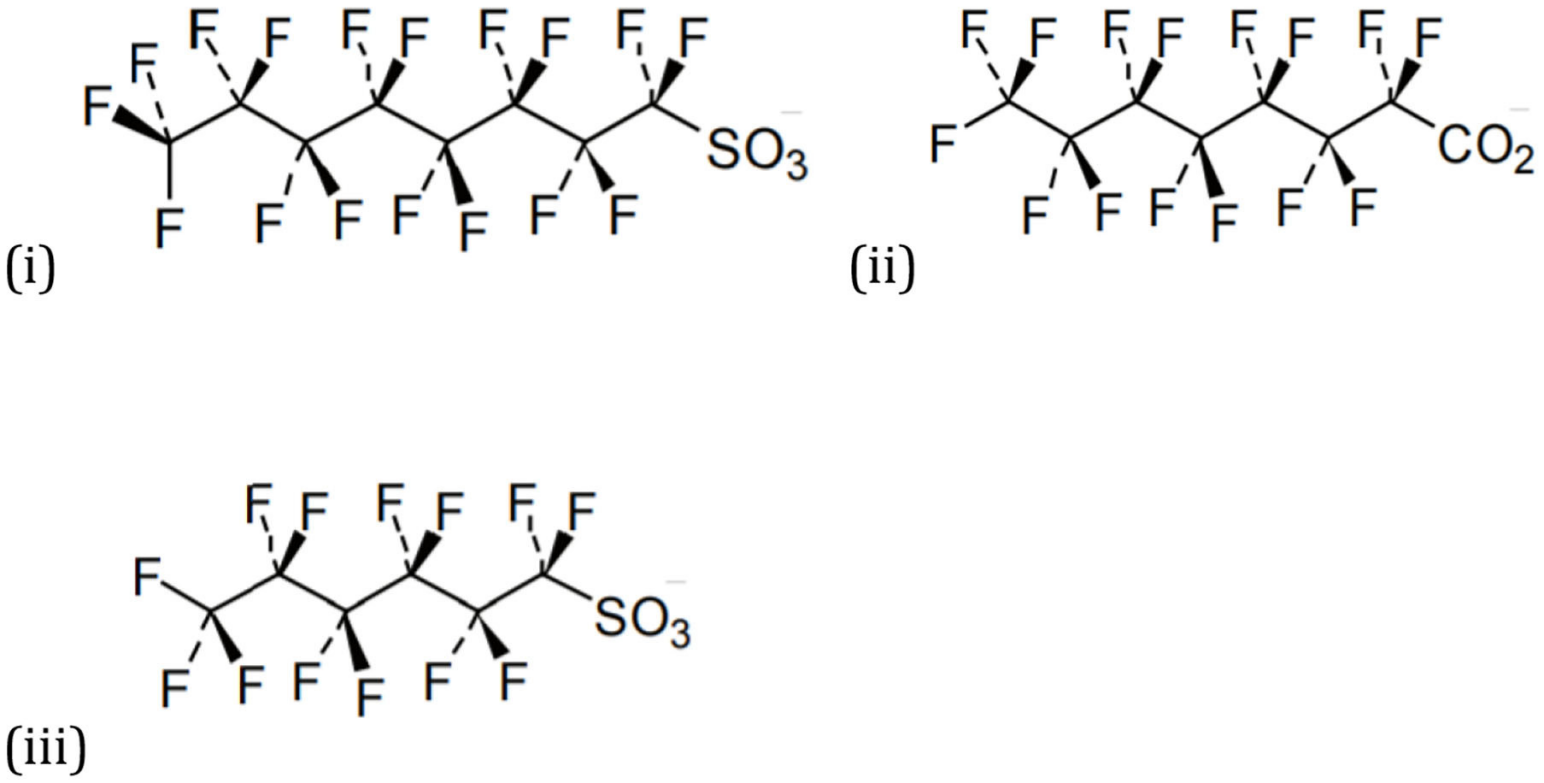
Structure of PFAS (i) PFOS (ii) PFOA, (iii) PFHxS structure (Buck et al., 2011).

Per-and polyfluoroalkyl substances (PFAS) are a group of synthetic man-made compounds manufactured for their ability to interact between two immiscible fluid phases acting as a surfactant (Buck et al., 2011; Rahman et al., 2014). PFAS are highly polar and contain strong carbon-fluorine bonds (C-F) which display unique amphiphilic properties (Figure 1). Generally, most PFAS exhibit (i) high thermal resistance, (ii) high chemical stability, and (iii) resistance to biotic degradation (Buck et al., 2011; Lindstrom et al., 2011; Rahman et al., 2014). Two broad categories of PFAS have been defined:

- ***Perfluoroalkyl substances*** typically comprise of short and long carbons chains (C2-C13+) and have a charged functional group head which is attached to one end. Generally, this functional group will be a carboxylic or sulfonic acid. Fluorine atoms attach to all bonding sites on the carbon chain except for the last carbon group head forming multiple carbon-fluorine (C-F) bonds (Figure 1). C-F bonds have the dissociation energy of 450 kJ mol^−1^ compared to carbon-chlorine and carbon-bromine bonds at 330 and 194 kJ mol^−1^, respectively (Parsons et al., 2008).
- ***Polyfluoroalkyl substances*** are not fully fluorinated. These substances have at least one lapse in the chain which is not a fluorinated atom—typically hydrogen or oxygen—which attaches to one of the carbon-chain tails. Polyfluoroalkyl chains contain carbon-hydrogen (C-H) bonds which create weak chains that are susceptible to biotic or abiotic degradation (Buck et al., 2011).

Three specific PFAS compounds, perfluorooctane sulfonate (PFOS), perfluorooctane acid (PFOA) and perfluorohexane sulfonate (PFHxS) (Figure 1) are known to have been contained in much older Aqueous Film Forming Foams (AFFF). Within environmental pH values, both PFOA and PFOS exist as anions (Rahman et al., 2014). Generally, PFAS do not degrade in water or soil under normal conditions, although it is thought that they can undergo physiochemical changes and breakdown into smaller alkyl chains (Teaf et al., 2019). The physicochemical properties of these three major PFAS compounds are presented in Table 1 and further discussed below:

- ***Perfluorooctane Sulfonate (PFOS)*,** CAS number 1763-23-1, is a completely fluorinated compound that contains eight carbon atoms and a sulfonate group head. PFOS has been used as coatings and protectants. PFOS is produced commercially from perfluorooctanesulfonly fluoride (POSF) which was used as an intermediate to produce other fluorochemicals. PFOS is formulated by C_8_HF_17_O_3_S_1_ which has a molecular mass of 500.13 g/mol. PFOS is produced through Simons Electro-Chemical fluorination (SECF), which produces 70% linear chains and 30% branched chains isomers. PFOS can also be created through telomerization which produces linear chains. As PFOS has major impurities in the form of other POSF-derived fluorochemicals it can be formed in the environment through the degradation of POSF-based products (Buck et al., 2011).
- ***Perfluorooctanoic Acid (PFOA)*,** CAS number 335-67-1, is a completely fluorinated organic acid with seven-carbon (C7) atoms and a carboxyl functional group head. PFOA has been a main constituent of AFFFs, as well as non-stick coats, and waterproofing. PFOA is formulated by C_8_HF_15_O_2_ which has a molecular mass of 414.07 g/mol (Buck et al., 2011).
- ***Perfluorohexane Sulfonate (PFHxS)*,** CAS number 355-46-4, has 6 carbons (C6) and is a completely fluorinated organic acid that is capable of repelling oil and water which have been used in the manufacturing of AFFF. PFHxS displays similar properties to both PFOS and PFOA. PFHxS is formulated by C_6_HF_13_O_3_S and has a molecular mass of 400.12 g/mol (Buck et al., 2011).

**Table 1:** Physiochemical properties of PFAS (Buck et al., 2011).

**Characteristic**	**PFOS**	**PFOA**	**PFHxS**
Appearance	White powder (potassium salt)	White to off-white powder	White crystalline powder
Melting point	>400°C (potassium salt)	54.3°C	No data
Boiling point	258–260°C	192.4°C	114.7°C >400°C
Density	~0.6 (potassium	1.7292 g/mL at 20 °C	1.84 g/mL at 20°C
Water solubility	519 mg/L at 20°C; 680 mg/L at 24–25°C	Soluble, 9.5 g/L at 25°C	Slightly soluble
Organic solvent solubility	56 mg/L	Soluble in polar organic solvents	No data
Log Kow	Not measurable	6.30 (estimated) in octanol-water mixture	Not measured
pKa:	−3.3 (estimated)	Debated; values of 2.8 and 3.8 have been reported. 0.5 has been estimated.	0.14

Based on the physiochemical properties, PFAS have been identified to have bioaccumulation potential, which tends to increase with increasing chain length. Most health research has been conducted on individuals with high levels of PFAS accumulated in their organs due to work on containment sites, airbases, and in response firefighting. A significant number of PFOA-related chronic diseases which include kidney and testicular cancers, ulcerative colitis, high cholesterol have been reported among PFAS-exposed individuals (Darrow et al., 2013; Steenland et al., 2013; Starling et al., 2017; Sunderland et al., 2019). PFOS and PFOA are readily absorbed through the gut and are not metabolized, meaning body loads become excessive before they are excreted. PFAS are believed to act as endocrine disruptors through the alteration in estrogen- and androgen-receptor functions (Mora et al., 2017). Human exposure to PFAS, produced by industry, occurs through ingestion of contaminated drinking water, food and household dust, inhalation of indoor air, and contact with other contaminated media (Trudel et al., 2008). Drinking water sources include rivers, lakes, and ground water, which can all be contaminated from industrial sources. In addition, there appears a significant exposure risk from contaminated treated sewage sludge (biosolids), as fertilizer, and recycled water from wastewater treatment plants, which are often used in agriculture, providing potential human exposure through contaminating crop foods (Sunderland et al., 2019).

## Sources of Contamination of PFAS and Fate in the Environment

The following section describes major sources of PFAS in the environment.

### Aqueous Film Forming Foams (AFFFs)

Aqueous Film Forming Foams (AFFFs) are intended to be used on flammable liquid fires through the process of combining hydrocarbon foaming agents with fluorinated surfactants when mixed with water (Backe et al., 2013). This creates interfacial tension that spreads across the surface of a hydrocarbon fuel, which extinguishes the flame, and forms a vapor barrier between the fuel and atmospheric oxygen, preventing re-ignition (Backe et al., 2013; Weiner et al., 2013; Harding-Marjanovic et al., 2015). The fluorotelomer AFFF, although not directly made with PFOA, and therefore less toxic to the environment has precursors that breakdown into PFOA in the natural environment (Backe et al., 2013; Weiner et al., 2013; Harding-Marjanovic et al., 2015). Typically, fluorotelomer based AFFF contains short-chain (C6) PFAS which can range from 50 to 98% short chains balanced with long-chain PFAS which can break down to PFOA.

Fire training facilities undergo extensive and prolonged use of AFFFs, which has caused large volumes of PFAS to be released into adjacent soils during short periods (Dauchy et al., 2019). From there PFAS leaches into groundwater supplies. Soils that do not contain high amounts of Total Organic Matter (TOC) through a lack of vegetation, land clearing, and anthropogenic abuse make it difficult for the chemicals to bind within the soil substrate (Allred et al., 2015; Gallen et al., 2018). Dauchy et al. (2019) sampled 44 soil cores and 17 groundwater samples from a firefighting drill sites active for more than 30 years and detected PFOS, 6:2 FTSA and 6:2 FTAB as the most predominant PFAS in surface soil; the highest total concentrations detected were 357 μg g^−1^, despite the presence of clay layers. However, the highest total PFAS concentrations were detected in the wells at the perimeter of the firefighter training site as well as the spring located downgradient of the groundwater flow. These concentrations ranged from 300 to 8,300 ng L^−1^; 6:2 FTAB was detected in water table 20 m belowground indicating these chemicals are not contained.

### Landfill Leachate

Landfills are designed to undergo large amounts of decomposition from natural and man-made organic compounds. Realistically, landfill serves as temporal and spatial storage. In a landfill, soil chemistry is heavily compromised which impacts natural degradation processes due to the number and nature of pollutants present. PFAS within waste can become mobile and leach into pore water creating contaminated leachate. Fortunately, modern sanitary landfills typically have stringent mechanisms for preventing and mitigating leachate from entering groundwater. However, the controlled discharge of leachate to wastewater treatment plants is allowed. Reinforcement of smaller and older sites to stop the threat of local point source contamination into surrounding soil and groundwater is paramount. PFAS will continue to persist in the landfill and continue to increase over time (Gallen et al., 2018). Studies examining landfill leachate confirmed that PFHxS was detected at high concentrations (mean 1,700 ng L^−1^; range 73–25,000 ng L^−1^); PFOA contamination was on average 690 ng L^−1^ (range of 17–7,500 ng L^−1^) and PFOS was detected at concentrations with a mean of 310 ng L^−1^ (range 13–2,700 ng L^−1^). Samples from sites with higher levels of PFAS profiles generally had greater proportions of construction and demolition waste. Dealing with landfill chemistry will require novel treatment pathways to deal with the existing PFAS loads on-site (Hamid et al., 2018).

Hepburn et al. (2019) stated that groundwater systems are at risk from increased urban re-development on former industrial land and this would lead to increased human exposure to PFAS. Their research indicates that legacy landfills are poorly constructed in major Australian urban developmental precincts. PFOS, PFHxS, PFOA, and PFBS were all detected in samples surrounding 13 sample locations including sites directly on waste material and down-gradient of landfills indicating evidence of leachate contamination. Many urban areas contain unknown amounts of legacy landfills which may lack any former leachate control, increasing the probability of contaminating local aquifers.

### Biosolids and Recycled Water

Point sources of PFAS transmission to agriculture occurs through the application of recycled water from wastewater treatment plants, landfill leachates and biosolids applied to agricultural land (Blaine et al., 2014; Ghisi et al., 2019). Venkatesan and Halden (2014) monitored soil amended with PFAS-containing biosolids over 3 years. They observed a loss of short-chain PFAS compounds within 100 days of application, due mainly to groundwater and surface water leaching. In a laboratory-based study by Allred et al. (2015) on the physical and biological release of PFAS from landfill leachate, they reported that increased leaching occurred from biological reactors under methanogenic conditions compared to abiotic reactors.

Once in agricultural lands, PFAS can be taken into the root systems of plants including cereals, fruits, and vegetables. PFAS with higher chain lengths are usually restricted to the roots, whereas shorter chains compounds can extend further (Ghisi et al., 2019). Generally, the physicochemical properties of the soil together with the plant uptake system will determine the rate and accumulation of PFAS; however, generally, PFOS accumulates at greater concentrations compared to PFOA. Pérez et al. (2013) showed that the PFAS in human tissue was 263 and 807 ng g^−1^ in the kidney and lung. In plants, the amount varied; however, most experiments used the spike method for contamination of soil. For example, Stahl et al. (2009) showed that ryegrass accumulated PFAS ranged between 408 and 7,520 μg kg^−1^ dry weight when the soil was contaminated with 0–50 mg kg^−1^ PFAS.

### PFAS in Soil Systems

As a consequence of these major sources of PFAS, these compounds are almost ubiquitously detected in the environment (Xiao et al., 2015; Lu et al., 2018). Research has indicated that soil organic carbon content is the dominant solid-phase parameter which affects the adsorption of PFAS. Solid matrices influence the environmental fate of hydrophobic organic contaminants (Higgins and Luthy, 2006). However, the different behavior of PFAS in comparison to traditional ionisable organic pollutants is due to their hydrophobic and hydrophilic functionalities (Li et al., 2018). Adsorption to soil or sediment can occur through two-mechanisms: interaction of their hydrophobic fluorinated carbon tails with the organic carbon fraction of the soil, or to a lesser extent by electrostatic interactions of the polar head group with the charged clay fraction (Kucharzyk et al., 2017). Longer-chained PFAS appear to sorb to soils more readily. PFAS with sulfonate groups sorb more than carboxylates. In comparison to PFOS which has a higher sorption capacity, PFOA is usually found in the dissolved phase. Perfluorinated acids appear to bind to soils with higher total organic carbon (TOC) and iron oxide concentrations; Li et al. (2018) achieved an adsorption equilibrium in ~48 h. Their results indicated that both PFOS and PFOA adsorption are influenced by TOC, proteins and saccharides. Similarly, iron and aluminum oxides also appear to be key parameters for adsorption of PFAS. Some forest soil vegetation shows greater ability to accumulate PFAS; the background levels of PFOA and PFOS in 28 forest soils suggested that PFOA concentrations were greater in precipitation at higher altitudes (Cabrerizo et al., 2018). In contrast, the concentration of PFOA in temperate grasslands appears to be much lower (Wang et al., 2018).

In summary, both bioaccumulation and translocation of PFAS occur from both natural terrestrial and aquatic environments and anthropologically built-up areas (Giesy and Kannan, 2001, 2002; Giesy et al., 2010; Xiao et al., 2012; Hu et al., 2016; Hepburn et al., 2019). The distribution of PFAS is enhanced by leaching and discharge into adjacent locations from treatment plants and urbanized redevelopments; eventually reaching oceans, including the North Pacific and the Arctic Ocean (Cai et al., 2012; Hepburn et al., 2019). However in addition, PFAS are now thought to be able to travel through airborne particles and wet and dry atmospheric deposition (Nakayama et al., 2019).

There is only limited information regarding the fate of PFAS in the environment. This is in part due to the difficulty associated with the detection of PFAS in the environment. Avoiding cross-contamination in the sample is difficult due to ambient atmospheric contamination. Most materials will at some point directly come in contact with fluorocarbons (Nakayama et al., 2019).

## Remediation Approaches

Technological approaches looking at the removal of PFAS from waste streams or contaminated environments tend to be expensive or impractical for the *in situ* removal of the contamination (Kucharzyk et al., 2017) (Table 2). Energy-intensive methods, such as high pressures and temperatures can disrupt and harm the balance of delicate ecosystems. Non-energy-intensive technologies such as granular activated carbon adsorption, sonolysis (generating chemical reactions using an acoustic field in a solution) and reverse osmosis have all shown some potential application for PFAS removal during field studies (Kucharzyk et al., 2017; Sorengard et al., 2019). Unfortunately, however, most treatment methods appear to collect rather than dismantle the C-F bonds, resulting in a residue containing PFAS that inevitably needs to be placed in a landfill.

**Table 2:** Removal technologies of PFAS from the environment.

**Technologies**	**Process**	**Site**	**Advantages**	**Disadvantages**	**Source**
Adsorption	Removal of PFAS compounds *via* adsorption to selective materials of adsorbing potential (e.g., Biochar, Resin, and modified clays)	*Ex situ/in situ*	Low operational cost and uses several materials which are commercially available	Ineffective for short-chain PFAS removal Interfere with other pollutants May require a large quantity of the adsorbent may be required, which causes a change in the land use.	Zhang et al., 2011
Filtration	Uses Reverse osmosis or Nanofiltration to remove PFAS compounds	*Ex-situ*	Effective under a wide range of pH	Expensive PFAS molecular weight dependant Creates high concentration waste	Tang et al., 2007
Thermal	Vaporizing the contaminants through increasing temperature to about 600 −1,000°C.	*Ex situ*	High destruction potential of the PFAS compounds	Time-consuming, high-cost and energy-intensive approach. Disturbs the soil and the ecosystem.	Yamada et al., 2005
Chemical oxidation/ reduction	Using chemical oxidants/reducing agents for the abiotic breakdown of contaminants	*In situ and ex situ*	Potential for PFAS mineralisation; effective in PFOA removal	Very expensive as it requires a large volume of chemicals and centralized equipment. Not applicable to treat all PFAS compounds. Short-chain PFAS could result. Interferes with other contaminants.	Yates et al., 2014; Arvaniti et al., 2015
Soil washing	Detaching PFAS from the soil by washing with water	*Ex situ*	Requires low technology Land reuse could be possible.	Expensive and time-consuming. Contaminated water results.	de Bruecker, 2015
Bioremediation	Use of biological agents (e.g., Microorganisms and Plants) to breakdown or accumulate PFAS compound	*In situ* and *ex situ*	Simple, cost-effective, and environmentally safe (Green) approach	Limited evidence that PFAS can be degraded. It could take a long time due to the slow biodegradation of PFAS.	Presentato et al., 2020

Several methods, including adsorption, filtration, thermal, chemical oxidation/reduction and soil washing have been developed for the removal of PFAS from environments. An outline of these approaches is shown in Table 2. Like all methods, there are both advantages and disadvantages related to each method. For example, soil washing is an *ex situ* technology which requires low technology input. However, it is expensive and may lead to water contamination (de Bruecker, 2015). These technologies have been thoroughly reviewed (Kucharzyk et al., 2017; Mahinroosta and Senevirathna, 2020). While some of these technologies have shown promising outcomes in laboratory-based studies, their cost-effectiveness, field applicability and feasibility are open to question (Mahinroosta and Senevirathna, 2020). Current commercial methods for remediating PFAS-contaminated environments, based on the use of one or more of the above treatments are only available for groundwater and not soils. There is therefore an urgent need to develop methods for the *in situ* bioremediation in the soil at sites contaminated by PFAS. Chemical and physical methods tend to be more expensive than bioremediation approaches, since bioremediation often treats contamination in place, allowing post-clean-up costs to be substantially reduced (Shahsavari et al., 2019).

Bioremediation, which is the use of a biological agent to breakdown contaminants, could represent a simple, environmentally safe and cost-effective technology to treat PFAS-contaminated soils. The commercial application of bioremediation has been successfully applied to remediate a variety of organic contaminants such as petroleum hydrocarbons, chlorinated substances and pesticides (Adetutu et al., 2015; Uqab et al., 2016; Khudur et al., 2019). However, the ability of biological agents to degrade PFAS is poorly studied (Kucharzyk et al., 2017).

### Bioremediation Options

Biodegradation of PFAS may involve enzymes that directly remove fluorine atoms from these compounds either (i) by adding oxygen across the F-C bond, i.e., oxidation, or (ii) adding electrons across the F-C bond, i.e., reduction, allowing other normal assimilation enzymes to breakdown the rest of the compound. The F-C bond is very strong and therefore difficult to destroy, which leads to its environmental stability. Therefore, it significant energy is required to catalyze reaction; biologically this can be provided *via* oxidative or reductive processes. There are known microbes that can break a F-C bond, either under aerobic or anaerobic conditions (Goldman and Milne, 1966; Tiedt et al., 2016, 2017); these are further discussed below.

It has been shown that some bacteria are able to bioaccumulate PFAS in aerobic and to a lesser degree, anaerobic conditions (Table 3); most of these bacteria have been identified as *Pseudomonas* sp. While there have been no confirmed reports of the biological removal of fluorine atoms from PFAS, the defluorination of monofluorinated compounds by many bacteria has been reported (Huang and Jaffé, 2019). For example, under aerobic conditions, pseudomonads have been isolated which can utilize fluoroacetate as a sole carbon source. In this case, the defluorination occurs through:

$$\begin{array}{l} {\text{FCH}}_{2}{\text{COO}}^{-}+{\text{OH}}^{-}>{\text{HOCH}}_{2}{\text{COO}}^{-}\\ \;+{\text{F}}^{-},\text{where the oxygen of the}\\ \text{ hydroxyl group is derived from water}. \end{array}$$

Indigenous bacterial species isolated from PFAS-contaminated environments have shown the ability to remediate PFAS compounds; two strains of *Pseudomonas* (PS27 and PDMF10) were able to remove 32 and 28% of PFAS compounds, respectively, within 10 days of incubation under alkanotrophic conditions (Presentato et al., 2020). Further, a decrease of around 32% in PFAS was also reported during a 96 h incubation of *Pseudomonas parafulva* (Yi et al., 2016) along with a 67% decrease in PFAS concentration over 48 h incubation of *Pseudomonas aeruginosa* (Kwon et al., 2014). In another study, *Pseudomonas plecoglossicida* utilized PFAS as an energy source, producing perfluoroheptanoic acid and releasing fluorine ions as a result (Chetverikov et al., 2017). A recent publication reported that following incubation of the ammonium oxidizing bacterium, *Acidimicrobium* sp. strain A6 with hydrogen as the sole electron donor for 100 days a 60% reduction in PFAS concentration was observed (Huang and Jaffé, 2019).

**Table 3:** Bacteria reported to be capable of bioaccumulating PFAS.

**Bacterial sp**.	**Process**	**Conditions**	**Concentration removed (%)**	**Initial PFAS concentration**	**Treatment time**	**Source**
*Pseudomonas* sp. strain PS27	Bioaccumulation	Aerobic	32	200 ng L^−1^	10 days	Presentato et al., 2020
*Pseudomonas* sp. strain PDMF10	Bioaccumulation	Aerobic	28	200 ng L^−1^	10 days	Presentato et al., 2020
*Pseudomonas parafulva*	Biodegradation	Aerobic	32	500 mg L^−1^	96 h	Yi et al., 2016
*Pseudomonas aeruginosa* strain HJ4	Biodegradation	Aerobic	67	1,400–1,800 μg L^−1^	48 h	Kwon et al., 2014
*Pseudomonas plecoglossicida* 2.4-D	Biodegradation	Aerobic	75	1 g L^−1^	6 days	Chetverikov et al., 2017
*Acidimicrobium* sp. strain A6	Defluorination/ biodegradation	Anaerobic	60	100 mg L^−1^	100 days	Huang and Jaffé, 2019
*Gordonia* sp. strain NB4-1Y	Biodegradation	Sulfur-limiting	70	n.d.	7 days	Shaw et al., 2019

While these studies focussed on relatively small organic compounds that contained fluorine atoms, they may help us to understand how selected microbes may break F-C bonds in PFAS. In these terms, it may be also useful to consider microbial activities known to break Cl-C bonds. For example, *Dehalobacter* sp. strain TeCB1 was able to carry out the reductive dechlorination of 1,2,4,5-tetrachlorobenzene to 1,3- and 1,4-dichlorobenzene with 1,2,4-trichlorobenzene being the intermediate daughter product (Alfán-Guzmán et al., 2017). A key enzyme is PceC, and the C subunit of the tetrachloroethene (PCE) reductive dehalogenase is encoded by the conserved pceABCT gene cluster identified in the microbial strain *Dehalobacter restrictus* PER-K23 (Buttet et al., 2018). Importantly, providing an electron donor can improve reductive dehalogenation catalyzed by specific bacteria (Holliger and Schumacher, 1994). To grow, these microbes utilize organohalide respiration (OHR), which is the energy metabolism of anaerobic bacteria able to use halogenated organic compounds as terminal electron acceptors (Buttet et al., 2018).

**Figure 2:**
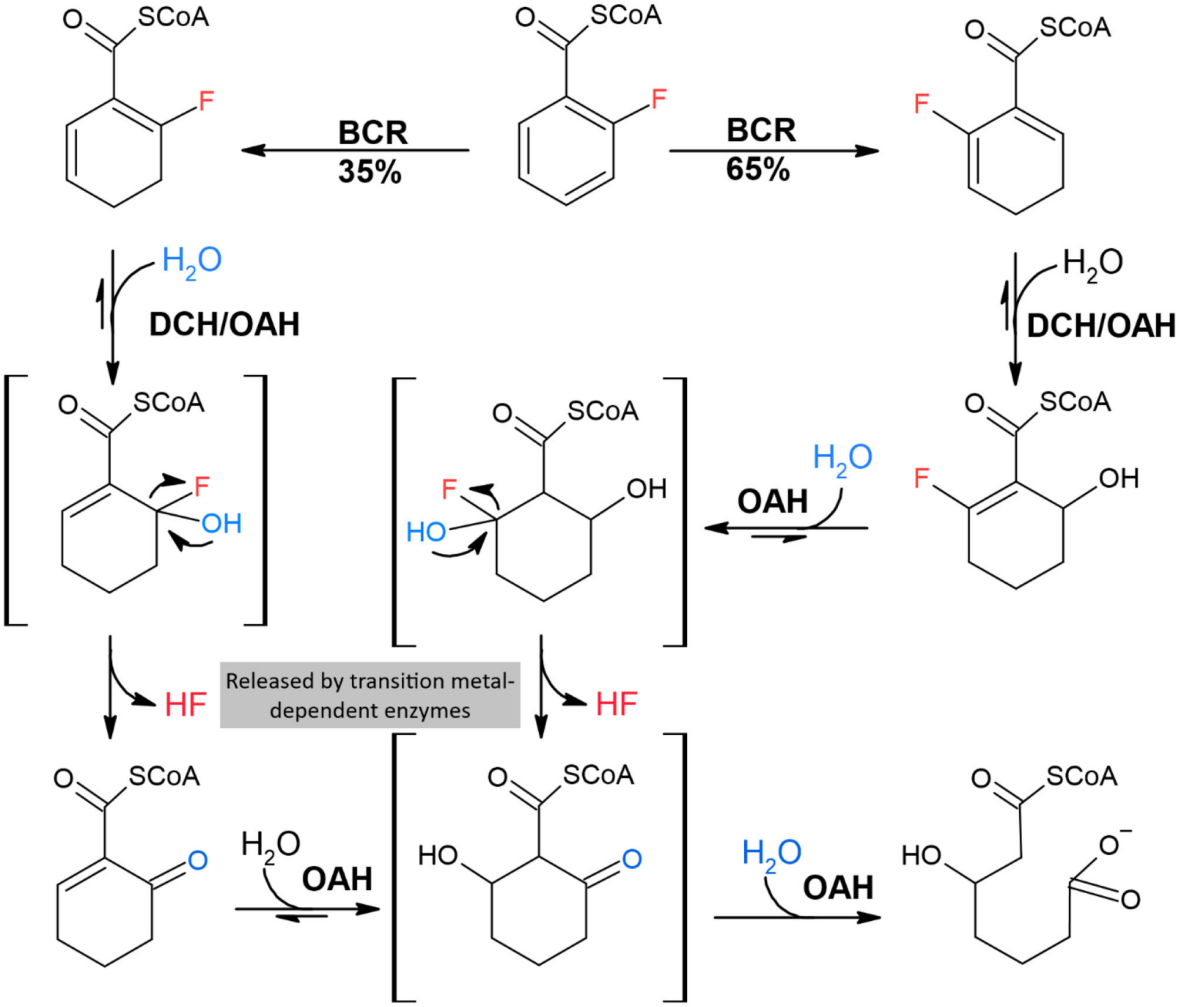
Proposed mechanism for biotransformation of 2-F-BzCoA by the microbial enzymes BCR, DCH, and OAH. The chemicals shown in brackets are likely to be unstable intermediate degradation compounds. Redrawn from Tiedt et al. (2017).

In terms of potential enzymes capable of degradading PFAS, reduction could be undertaken by a P450-type enzyme or similar. In organic chemistry F, in F-C bonds, can be replaced by transition metals (Kiplinger et al., 1994), and therefore transition metal-dependent enzymes can release F from F-C bonds (Figure 2). The F in F-C bonds is significantly electro-negative, and therefore can promote attraction to transition metal cations. The value of a transition metal in an enzyme reaction is to allow the recycling of its charge state. For example, P450-type enzymes contain a transition iron cation, with activity modified by a heme group for its reaction. Some mixed-function oxidases and horseradish peroxidases have been reported to defluorinate monofluorinated compounds (Goldman and Milne, 1966).

Recently a mode of oxygen-independent defluorination was identified for the complete degradation of para-substituted fluoroaromatics by the denitrifying bacterium *Thauera aromatica*. This microbe utilizes a class I benzoyl-coenzyme A (BzCoA) reductase (BCR), which catalyzes the ATP-dependent defluorination of 4-F-BzCoA to BzCoA. Other enzymes involved are 1,5-dienoyl-CoA hydratase (DCH) and bifunctional 6-oxo-1-enoyl-CoA hydrolase (OAH). The outcome of the complete degradation of 2-F-benzoate is the production of HF and CO_2_ (Tiedt et al., 2016, 2017) (Figure 2).

Both F-1,5-dienoyl-CoA isomers (compounds 2/2^*^) are hydrated to different F-OH-1-enoyl-CoA isomers (compounds 11/11^*^) by DCH and OAH, respectively. Unstable 6-F-6-OH-1-enoyl-CoA (11^*^) spontaneously decomposes to 6-oxo-1-enoyl-CoA (compound 7) by HF-expulsion. This, in the presence of OAH becomes immediately hydrated presumably to 2-oxo-6-OH-cyclohexanoyl-CoA (compound 12) before hydrolysis to 3-OH-pimeloyl-CoA (compound 8). Stable 2-F-6-OH-1-enoyl-CoA (compound 11) can also only be further hydrated by OAH, apparently to the unstable 2-F-2,6-di-OH-cyclohexanoyl-CoA intermediate, which spontaneously decomposes to compound 12 before ring hydrolysis by OAH. Intermediates illustrated with brackets probably only occur transiently (Tiedt et al., 2017).

### Microbial Interaction With PFAS

Perfluorinated chemicals are chemically very stable and metabolically either completely stable or barely biodegradable so that they can be classified as persistent substances (Von Der Trenck et al., 2018). However, investigations have inferred that limited biotransformation of these chemicals can occur in natural and industrial environments. It is, therefore, important to understand what biotransformation occurs in practice, given the variation in toxicity across the range of potential PFAS products that may be produced due to biotransformation. A further goal is to find organisms that can significantly remove fluorine atoms from these compounds to substantially reduce their toxicity and stability.

**Figure 3:**
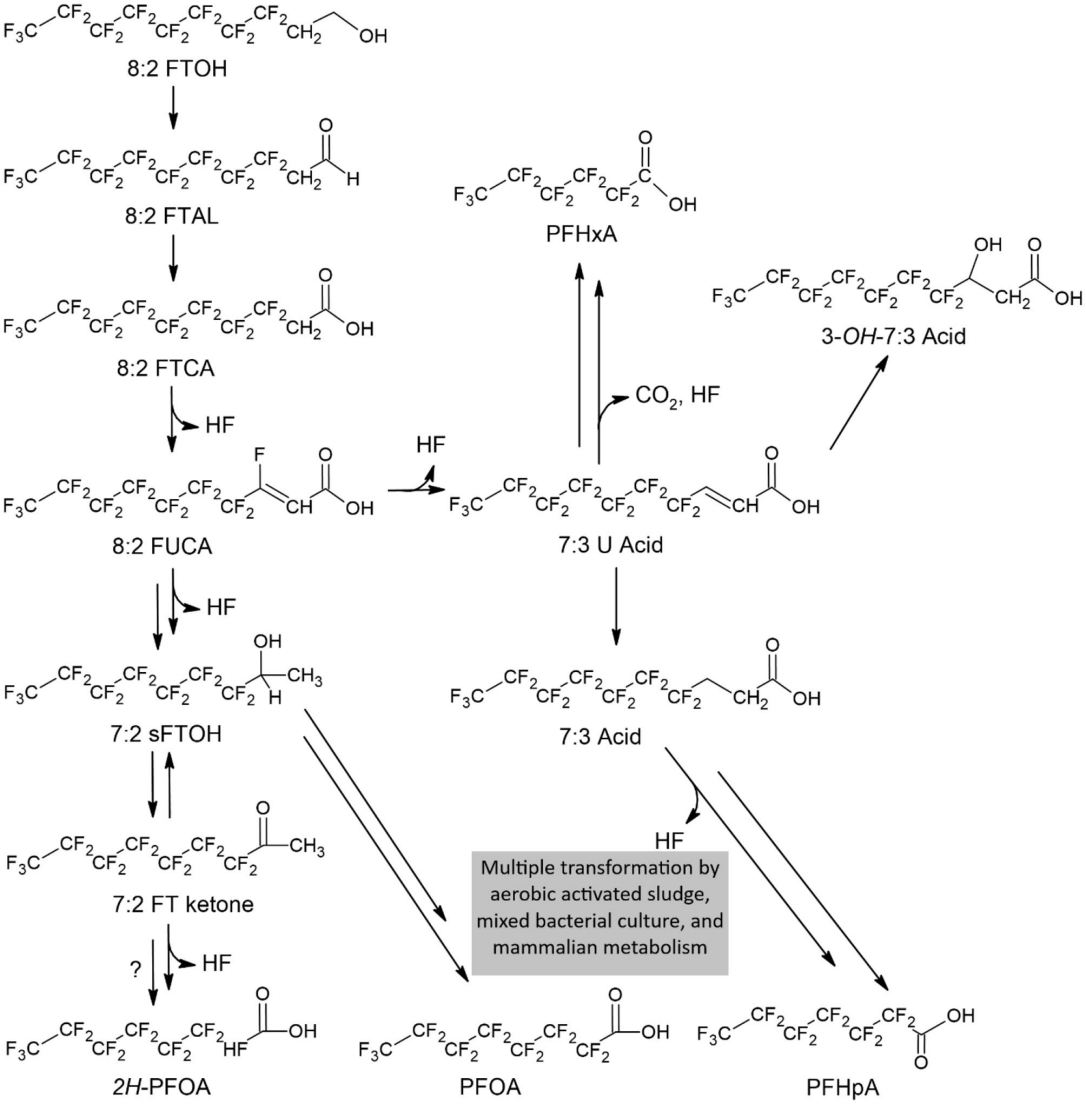
Proposed aerobic biodegradation pathways of the precursor 8:2 FTOH in soil. The double arrows indicate multiple transformation steps. Defluorination reactions are indicated by the release of fluoride ions (HF). Stable and semi-stable compounds are shown inside dashed boxes. 2H-PFOA has been proposed, but it has not been successfully validated as a PFOA degradation product Obtained. Redrawn from Liu and Mejia Avendaño (2013).

It has been demonstrated that fluorinated precursors can be transformed to PFAS, with variable efficiency by a range of biological systems (soil/wastewater; Liu and Avendano, 2013; Lee et al., 2014). Attempts have been made to infer pathways for the biotransformation of fluorinated precursors (D'eon and Mabury, 2007; Rhoads et al., 2008; Wang et al., 2009; Liu and Avendano, 2013; Lewis et al., 2016) (Figure 3). However, these tend to be limited to side chains, without significant removal of fluorine atoms. This is unfortunate, in terms of potentially increased toxicity, though may provide a useful basis to further investigate biodegradation of PFAS.

In a further study, two different microbial consortia were isolated from two river sediments in Saitama and Osaka, Japan, known for long term pollution with PFOS and PFOA (Beskoski et al., 2018). Amicrobial chemoorganoheterotrophic consortia which included bacteria, yeast and molds was able to significantly decrease concentrations of PFOS and PFOA between 46–69% and 16–36%, respectively. However, defluorinated PFOS and PFOA products were not detected, though several metabolites were found only in samples from consortia with PFOS and PFOA. It was suggested these were associated with unsaturated monofluorinated fatty acids and hydrocarbons with multiple unsaturated bonds or ring structures (Beskoski et al., 2018). Nevertheless, if confirmed, this is problematic as the fluorine is still attached to an organic molecule that could be simply transferred to other organisms through a food web.

While specific biodegrading pathways for some PFAS have been investigated (Liu and Avendano, 2013), for many PFAS, the pathways are unknown. Moreover, the types of enzymes and associated genes involved have not been reported. A degradation pathway for a particular PFAS may be investigated by assessing the intermediate products in a sampling time series. Organisms able to degrade these compounds may optimally be selected from environmental areas that have been polluted with the particular PFAS. This approach has been utilized for developing bioremediation of soils polluted with oil products (Moliterni et al., 2012). The types of enzymes involved in biodegradation may be inferred by assessing the intermediate products, followed by a search of the KEGG database (Kanehisa et al., 2018) to confirm the type of enzymes and infer the structural genes that produce these enzymes.

It is also important to understand the biodegrading pathways of PFAS precursors in more detail, to potentially support bioremediation of PFAS and their precursors. It is also useful to recognize that transformations of PFAS may be potentially caused by chemical factors, as well as biological activities.

While biotransformation of PFAS commonly occurs, high concentrations may reduce biotransformation rates due to chemical toxicity. The impact of PFOA on the activated sludge process has been assessed using a lab-scale sequencing batch reactor, which was continuously exposed to PFOA (Yu et al., 2018). This method used a representative concentration for PFAS (20 mg L^−1^) to mimic extreme conditions from industrial waste or groundwater from fire-fighting practice sites. The results indicate that PFOA restrained microbial growth which affected dissolved organic carbon removal. Also, continued exposure to PFOA resulted in a significant shift in community structure, leading to the presence of more PFOA-tolerant species (*Bacteroidetes, Proteobacteria*, and in particular *Acidobacteria*) (Table 3).

It is important to investigate biodegradation of PFAS under both aerobic and anaerobic conditions and in particular, to assess biotransformation intermediates in a reductive environment (Liu and Avendano, 2013), given the current limited PFAS biodegradation reported in aerobic environments. An oxygen-independent pathway may lead to enhanced degradation (Tiedt et al., 2016, 2017).

### Mycoremediation

To date, research is limited on their ability of fungi to degrade PFAS. This is perhaps surprising given they are known to degrade lignin, one of the most recalcitrant natural compounds along with many toxic natural and xenobiotic compounds including organochlorines [e.g., DDT and DDE, organophosphates, pesticides, including chlorpyrifos and polychlorinated biphenyls (Beaudette et al., 2000) andpolyaromatic hydrocarbons (Moghimi et al., 2017)].

White-rot fungi in particular have displayed relative success in terms of the biotransformation of organic toxicants, including polychlorinated biphenyls, organophosphate pesticides and polycyclic aromatic hydrocarbons (Kaur et al., 2016; Stella et al., 2017; Harry-Asobara and Kamei, 2019). There are very few studies examining their ability to degrade PFAS. Tseng et al. (2014) reported some promising preliminary results looking at the effects of wood-rotting fungus on 6:2 FTOH, using the ligninolytic fungi, *Phanerochaete chrysosporium. P. chrysosporium* was capable of transforming 50% of 6:2 FTOH and 70% 8:2 FTOH in 28 days. Major metabolites of 6:2 FTOH included 5:3 polyfluorinated acid (40%), 5:2 FTOH (10%), PFHxA (4%). In contrast, the non-lignolytic fungus *Aspergillus niger* was unable to transform 6:2 FTOH over 35 days. while the same study reported that *P. chrysosporium* was capable of transforming 20% PFOS within 28 days. However, this research was conducted in a laboratory; it remains to be determined whether ligninolytic fungi are capable of degrading PFAS in the environment.

### Phytoremediation

Phytoremediation represents another potential bioremediation approach for PFAS removal from contaminated environments due to the ability of several plants to bioaccumulate PFAS. Although PFAS are not extensively degraded during phytoremediation, bioaccumulation in plants creates a potential route for removal of PFAS from contaminated environments. Phytoremediation has been successfully used for the removal of several environmental contaminants including heavy metals and chlorinated substances (Huff et al., 2020).

Several plants have been used to accumulate PFAS. The wetland species *Juncus effuses* accumulated 11.4% of seven PFAS compounds from PFAS-spiked soil (Zhang et al., 2019). *Betula pendula* and *Picea abies* were reported to accumulate up to 97 and 94 ng g^−1^, respectively, during a study at a firefighting training site near Stockholm, Sweden, contaminated with 26 PFAS compounds (Gobelius et al., 2017). The phytoremediation of PFAS contaminated soils using herbaceous and woody plant species has also been reported (Huff et al., 2020). The potential of several plants in a greenhouse study to bioaccumulate 6 PFAS compounds over 14 weeks has been reported (Table 4).

**Table 4:** Bioaccumulation of PFAS compounds by herbaceous plant species [Adapted from Huff et al. (2020)].

**Plant species**	**Bioaccumulated PFAS compounds**											
	**PFPeA**		**PFHxA**		**PFOA**		**PFBS**		**PFHxS**		**PFOS**	
	**μg**	**%**	**μg**	**%**	**μg**	**%**	**μg**	**%**	**μg**	**%**	**μg**	**%**
*Amaranthus tricolor*	446	30.9	153	8.1	66	7.7	4	0.4	1	4	0	0
*Brassica juncea*	114	11.8	72	5.7	15	2.7	9	1.7	8	1.6	4	0.7
*Cynodon dactylon*	434	22.6	427	16.9	55	4.9	156	14.1	51	4.8	20	2
*Equisetum hyemale*	759	39.5	557	22.1	36	3.2	1	0.1	7	0.6	4	0.4
*Festuca rubra*	717	37.4	652	25.9	122	10.8	224	20.3	141	13.2	39	3.8
*Helianthus annuus*	52	5.5	8	0.6	4	0.8	2	0.4	3	0.6	1	0.2
*Schedonorus arundinaceus*	807	42	696	27.6	60	5.3	262	23.8	92	8.6	14	1.4
*Trifolium incarnatum*	29	3.1	11	0.8	50	8.9	13	2.3	10	1.9	1	0.2

### Future Prospects and Conclusion

Using a single bioremediation approach for PFAS may not be successful duet to the process is very slow therefore using a combination of bioremediation techniques to maximize the remediation of PFAS may offer a better approach (Ji et al., 2020). In one study, a combination of phytoremediation and PFAS-degrading bacteria in a constructed wetland was recommended as an effective and environmentally friendly approach that integrates optimum physio-chemical conditions and enhanced microbial degradation. The effectiveness of this “treatment train” approach has previously been reported in removing several emerging contaminants, such as pesticides, pharmaceutical and personal care products (Lv et al., 2016; Liu et al., 2019).

Constructed wetlands consist of three main components which are substrates, plants and microorganisms. The substrate, such as biochar, works as an absorbent of long-chain PFAS as well as media for plant growth and provides surface area for microbial biofilm production (Yang et al., 2018). Plants are another essential component of the constructed wetlands due to their ability to accumulate PFAS in different plant parts, including leaves and roots tissues (Zhang et al., 2019). Plant and substrate disposal, however, remains a great challenge. Thus, thermal treatment could be required for the complete mineralization of adsorbed and bioaccumulated PFAS (Gagliano et al., 2020). Microorganisms are the most important component of the wetland; however, the indigenous microbes have limited ability to biodegrade PFAS. The introduction of microorganisms that can degrade a certain contaminant has been proven to enhance the biodegradation of several emerging contaminants, such as antibiotics and personal care products (Li et al., 2019). Therefore, the introduction of defluorinating microorganisms that can use methane and hydrogen as an electron donor, to the constructed wetlands could enhance the breakdown of the C-F bond and the biodegradation of PFAS compounds (Huang and Jaffé, 2019). However, further investigation is required to assess the effectiveness of this approach (Ji et al., 2020).

Microalgae have shown the ability to remediate several emerging contaminants, including PFAS, through bioaccumulation, biodegradation and bio-adsorption. However, to date, most of the studies that have been conducted on microalgal-bioremediation are laboratory-based experiments under control conditions and the transition to field applications remains a challenge. Therefore, further research is required to employ microalgal species to bioremediate PFAS, which demonstrate increased biodegradation potential (Sutherland and Ralph, 2019).

The other role that microalgae may play in remediating emerging contaminants is enhancing bacterial biodegradation. Microalgal cells provide oxygen, an essential electron acceptor, *via* photosynthesis for the aerobic bacterial species, which in turn, produces CO_2_ which is required for microalgal photosynthesis (Sutherland et al., 2015). Microalgae release dissolved organic matter (DOM) which can biostimulate bacterial degradation of the contaminants although the mechanism for the bacterial biostimulation is not fully understood. Thus, investigating the relationship between microalgae and bacteria and the optimum physico-chemical conditions are crucial steps to enhance the bioremediation process (Sutherland and Ralph, 2019).

Both fungal and bacterial strains have been isolated that are capable of degrading PFAS; however, degradation is slow and incomplete. In addition, information regarding the biodegradation and bioaccumulation of PFAS using bacteria and fungi is limited. Thus, more research needs to be undertaken. This is a crucial limitation to the development of any robust bioremediation strategy. However, with the current array of approaches and tools available to microbial ecologists, including stable isotope probing, metagenomics, transcriptomics and metabolomics, the identification of degradative pathways and the subsequent harnessing of PFAS-exposed microbial communities for remediation remains a possibility and further work needs to be performed to underpin the degradation process. Further studies could lead to an understanding of the pathways of degradation, by comparison to proposed published pathways in soil, for example (Liu and Avendano, 2013). In turn, the possible enzymes involved in degrading PFAS can be inferred by comparing the structures of PFAS in the proposed pathways. It would also be of value to list the potential genes in key bacteria that express the types of enzymes involved in degrading It would be useful to find and characterize microbes in contaminated soils that are capable of degrading PFAS and to quantify bioaccumulation and biomagnification of PFAS in trophic levels of marine ecosystems, in particular, to improve the assessment of health risks in human consumption of seafood contaminated by PFAS. Stable isotope probing (SIP) has been an extremely useful tool to link microbial identity to function; this technique has been used to elucidate the microbes responsible for the degradation of a variety of xenobiotics (Dumont and Murrell, 2005). The technique can be performed with DNA-SIP (Uhlík et al., 2009) or RNA-SIP which has been used during the degradation of benzene (Aburto, 2007; Aburto and Ball, 2009) and naphthalene (Huang et al., 2009) in groundwater, phenol in a bioreactor (Manefield et al., 2002), phenol in sludge (Sueoka et al., 2009) and tetrachloroethene in river sediments (Kittelmann and Friedrich, 2008) among other types of studies involving trophic interactions, biogeochemical processes or ecosystem functioning (Gutierrez-Zamora and Manefield, 2010). More recently it has been used to identify pesticide degraders (Jiang et al., 2018) and 1-4 dioxane degraders (Aoyagi et al., 2018). Therefore, SIP is a powerful technique that can also be combined with metagenomics (Vo et al., 2007) and transcriptomics (Lueders et al., 2016) and could also aid during the bioremediation of PFAS.

Metagenomics is a valuable tool that has been used recently to assess the stress of polyfluorinated alkyl substances on the microbial community (Cai et al., 2020) as well as their dynamics and structure (Zhang et al., 2020) in different environments such as soil and freshwater ponds. It is also one of the tools of synthetic biology (synbio) which has been recently proposed to help in the bioremediation of xenobiotics, among them PFAS (Rylott and Bruce, 2020). Systems biology and protein design will also be critical tools for synbio, that in the future should allow the synthesis of proteins by reprogramming the genetic code and aid in the remediation of the persistent contaminants (Rylott and Bruce, 2020).

## Author Contributions

ES, DR, and LK wrote the manuscript. DT and AA-M contributed to the collection of literature and summarization. ES and AB guided throughout the preparation of the paper, proofreading the paper, and revised it. All authors fully agreed for publication of the paper.

## Conflict of Interest

The authors declare that the research was conducted in the absence of any commercial or financial relationships that could be construed as a potential conflict of interest.
